# Inter- and Intra-Population Variation of Foliage Calcium and Magnesium in Two Chinese Pine Species

**DOI:** 10.3390/plants12030562

**Published:** 2023-01-26

**Authors:** Meng Hu, Yuan Yang, Mingyang Fan, Kexin Huang, Lu Wang, Ting Lv, Xiangui Yi, Lin Chen, Yanming Fang

**Affiliations:** 1 Co-Innovation Center for Sustainable Forestry in Southern China, Key Laboratory of State Forestry and Grassland Administration on Subtropical Forest Biodiversity Conservation, College of Biology and the Environment, Nanjing Forestry University, Nanjing 210037, China; 2 College of Forestry, Nanjing Forestry University, Nanjing 210037, China

**Keywords:** needles, calcium and magnesium, variation

## Abstract

Calcium and magnesium are major nutrient elements of plants, and both play an important role in plant growth and development. *Pinus massoniana* and *P. hwangshanensis* are important afforestation tree species in barren mountains in China. However, observation and research on calcium and magnesium nutrition of dominant forest species in China are still limited. This study determined the concentration of calcium and magnesium in needles for two species from five sites in East China by inductively coupled plasma optical emission spectrometry (ICP-OES). We then explored the inter- and intra-population variation pattern of calcium and magnesium and their relationship with environmental factors. There were significant differences in traits among populations. The strongest factors, which impacted the variation of calcium and magnesium concentration, were elevation and individual differences, respectively. Element concentration was correlated to environmental factors such as longitude, latitude, elevation, and mean annual temperature. The results of this study can be helpful for a better understanding of tree growth, population survival, and forest succession.

## 1. Introduction

Calcium (Ca) and magnesium (Mg) are among the macronutrients of plants [1,2,3]. Based on principal component analysis (PCA) of elements, leaf chemical variation in nitrogen (N) and phosphorus (P) concentrations was recognized as the first axis of the “leaf economics spectrum” (LES) [4,5,6], whereas a gradient in Ca and Mg concentrations were labeled as the second axis of “structure and photosynthetic activity” [7], “mechanical properties” [8], or “digestibility” [9]. Thus, the variation pattern of plant Ca and Mg concentrations, although less understood than that of N and P, is crucial for understanding the ecological processes and functions of plants.

As one of the important elements in plant growth and development, Ca is associated with the cell wall structure and affects the plasticity and rigidity of the cell wall [10,11]. It is also involved in the stabilization of membranes, and a low concentration of free Ca^2+^ must be maintained in the cytoplasm to avoid the formation of insoluble calcium salts [2]. When deficient in Ca, plants may display meristem death, root disintegration, stem collapse, and leaf deformity and necrosis [3]. In addition, Ca is also a signal molecule in abiotic stress responses [12,13], biotic interactions [14], and pathogen defense [15]. Like Ca, Mg is also an exchangeable cation. Mg is supported to maintain the structural integrity of chlorophyll, ribosome, and chromatin [16,17]. It plays very important physiological roles in plants in photosynthesis and carbohydrate metabolism [18], as well as in the hydrolysis of high-energy compounds, and affects the activity of some enzymes such as DNA and RNA polymerases, ATPase, PEP carboxylase, and RuBisCO [19,20]. In the absence of Mg, plant leaves may show interveinal chlorosis, anthocyanin pigmentation, and then necrotic spotting [3,21].

In tree species, Ca and Mg are essential for tree metabolism and various physiological processes related to growth [22,23]. Cambial Ca^2+^ concentrations determine cell division, cell wall chemistry, and cell differentiation and then affect cambium width, vessel size, and wood formation [22]. Efficient Ca^2+^ supply may be a decisive factor in wood formation [24], which has significant impacts on assimilate partitioning and mineral distribution of the phloem [25] and lignification reaction in the wood [26]. Ca or a combination of nutrients (including Ca) may affect stem sinuosity [27]. In sapwood and heartwood, the Ca chemical species are distinct [28]. Thus, it can be served as a chemical marker for wood traits (e.g., wood anatomy and density) [29,30]. In addition, Ca:Mn ratios can be used as early-warning signals of forest dieback [31], and Ca physiology is considered to associate with the structure and function of forest ecosystems [32]. In terms of tree growth and development, the physiological function of magnesium is also essential. In *Pinus radiata*, Mg deficiency markedly reduces its net photosynthetic rate, biomass, and height growth [33,34,35]. Mg addition could reduce the symptom of extreme Mg deficiency and stimulate shoot growth in European beech [36]. Mg treatments may adjust the wood stiffness of standing trees in radiata pine [37]. In general, effective and balanced calcium and magnesium are very important for the function of the forest ecosystem [38]. Unfortunately, little is known about the plasticity level and the intra-specific variation patterns of calcium and magnesium nutrition in naturally growing trees and only a few reports have been found [39,40,41,42,43]. However, these data are not only necessary for nutrition diagnosis, fertilization scheme formulation, and plantation management for a forest stand, but also critical for tree genetic improvement and selective breeding.

*Pinus massoniana* (Masson pine) and *P. hwangshanensis* (Huangshan pine) are two very important tree species in southern China that belong to Subgen. *Pinus*, Sect. *Pinus*, and Subsect. *Pinus* [44]. The horizontal ranges of Masson pine (27°55′~32°30′ N, 102°40′~109°55′ E) a nearly sympatric to Huangshan pine (22°48′~31°48′ N, 112°30′~122°30′ E), with a lower elevation (generally below 700 m) in the former and a higher altitude (generally above 700 m) in the latter [45,46,47]. The two pine species share or own independently the following features: (1) in the same Subsection systematically; (2) with an overlapping range horizontally and distinct distributions vertically; (3) with different vegetation types, e.g., ‘Masson pine warm evergreen coniferous forest’ and ‘Huangshan pine cool evergreen coniferous forest’ [48,49]. Investigating the Ca and Mg nutrition of Masson pine, and Huangshan pine is undoubtedly essential for understanding their ecological processes, such as forest growth and development and nutrient cycling, as well as forestry practices, such as afforestation and forest management. Unfortunately, reports in this regard are limited [50,51]. A systematic and complete understanding with regard to the law of element variation, phenotypic plasticity, and ecological adaptability of the two species is still lacking. The knowledge about the effects of genetics and environment on elemental chemistry is also insufficient.

In this study, we aimed to solve the following three questions: (1) are there any differences in the variation levels and patterns of Ca and Mg nutrition between the two pines? (2) What are the plasticity level and variation pattern of Ca and Mg elements in each pine species? (3) What is the relationship between Ca and Mg concentrations and environmental factors? We expect that exploring the intra-specific variation of Ca and Mg nutrition of the two pines will provide solid data support for enriching the adaptive ecological theory and selective breeding practice.

## 2. Results

### 2.1. Difference in Ca and Mg Concentrations among Populations of Two Pines

The concentrations of Ca and Mg in *P. massoniana* and *P. hwangshanensis* were distinct (Table 1). Generally, the concentration of Ca was higher than that of Mg. The Ca concentration of *P. hwangshanensis* was significantly lower than that of *P. massoniana* in population S (population from Mt. Sanqingshan). As for Mg concentration, *P. massoniana* was significantly higher than *P. hwangshanensis* in population H (population from Mt. Huangshan), population J (population from Mt. Jinggangshan), and population T (population from Mt. Tianmushan).

Variation of element concentrations among populations also occurred within a species. For *P. massoniana*, the highest mean values in Ca concentration (5.62 mg·g^−1^) and Ca: Mg ratio (5.20) appeared in population S, whereas the top Mg concentration (1.41 mg·g^−1^) was in population J. Population L (population from Mt. Lushan) had the lowest mean Ca concentration (2.59 mg·g^−1^) and Ca:Mg ratio (2.47), while population H held the minimal mean Mg concentration (0.92 mg·g^−1^). For *P. massoniana*, the Ca concentration in population S was significantly higher than in other populations except for population H. Besides, population H had a significantly lower Mg concentration than population J and population T. The Ca:Mg ratio in population H and population S was significantly higher than that in population J, population L, and population T.

For *P. hwangshanensis*, population H had the highest mean Ca concentration and Ca:Mg ratio, with values of 3.78 mg·g^−1^ and 5.65. Moreover, the mean Mg concentration from population J was the highest, 1.09 mg·g^−1^, among different populations. The lowest mean Ca concentration and Ca:Mg ratio were in population J, with the values of 2.85 mg·g^−1^ and 2.60. Furthermore, the lowest mean Mg concentration occurred in population H (0.67 mg·g^−1^). In a word, the most abundant mean Mg concentration was in population J, and the lowest was in population H, no matter which species. Whether *P. hwangshanensis* or *P. massoniana*, the richest and the poorest mean Ca concentration and Ca:Mg ratio were in the same population. The Ca concentration of *P. hwangshanensis* had no significant difference among different populations. For *P. hwangshanensis*, population H held a significantly lower Mg concentration than population J and population L. Population J held a significantly higher Mg concentration than population T and population H. For Ca:Mg ratio, population H was significantly higher than other populations, and population T was significantly higher than population J and population L. 

The Ca and Mg concentrations varied in different populations to various extents, according to CV. For *P. massoniana*, the most changeable Ca and Mg concentrations were in population J, with the CV of 56.08% and 26.85%. And for *P. hwangshanensis*, the most changeable Ca and Mg concentrations occurred in population H and population L separately.

### 2.2. Correlation between Calcium and Magnesium

We found that there was a certain correlation between Ca and Mg concentrations partly. The correlation coefficient of *P. massoniana* of population L was the highest and most significant. It could be seen in *P. hwangshanensis* only the correlation coefficient of population J was significant (Table 2).

### 2.3. Source of Variation

**Figure 1:**
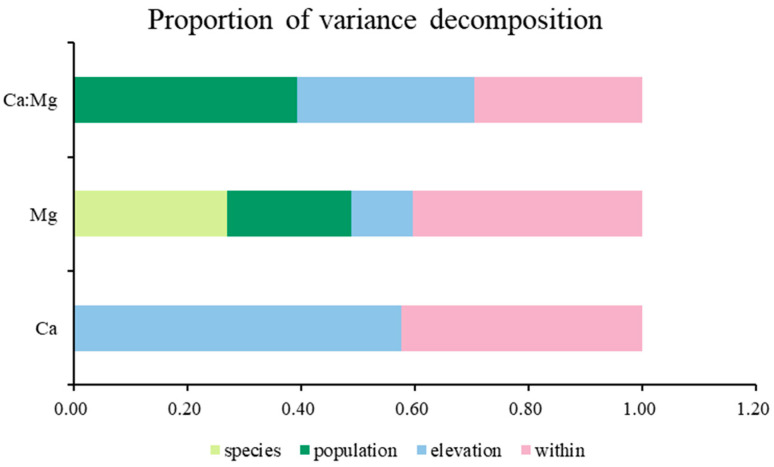
Proportion of variance decomposition. Different color block length represents the interpretation rate of different factors to variation of traits. Vertical axis represents different traits, and horizontal axis represents different proportion.

Figure 1 shows a partitioning of the variance to species, population, elevation, and among individuals (within). For Ca concentration, elevation accounted for approximately 0.58, and the value of the effects of population and species were close to zero. A little less than half the variance of the dataset was attributable to among individuals, which meant individual differences within the plot. Additionally, for Mg concentration, the principal source of variation was among individuals. Species, population, and elevation accounted for 0.27, 0.22, and 0.11 of the total variance, respectively. Otherwise, for Ca:Mg ratio, population, elevation, and among individuals accounted for approximately 0.39, 0.31, and 0.30, respectively. Partitioning of the variance to species for Ca concentration and Ca:Mg ratio were both not obvious in the figure but still had a very low proportion.

### 2.4. Correlation with Environmental Factors

**Figure 2:**
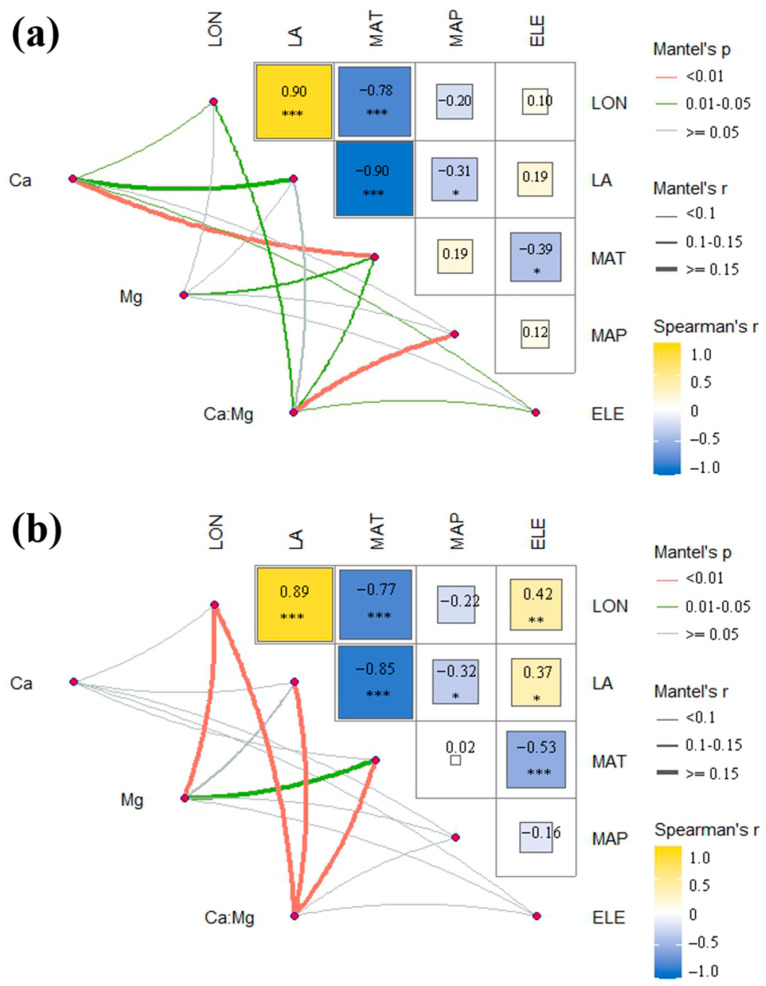
Correlation with environmental factors of two species. (**a**) *P. massoniana*; (**b**) *P. hwangshanensis*. Pairwise comparisons of environmental factors are shown with a color gradient denoting Spearman’s correlation coefficients. * *p* < 0.05, ** *p* < 0.01, *** *p* < 0.001. The different colors of lines represent the significance between traits and environmental factors. The correlation coefficients between traits and environmental factors are expressed by lines of different thickness.

There were obvious correlations between environmental variables (Figure 2). Mean annual temperature (MAT) was found to be negatively significantly correlated with longitude, latitude, and elevation. For *P. massoniana*, Ca concentration was strongly correlated with latitude and MAT. Mg concentration had no significant correlation with all environmental factors except MAT. Additionally, mean annual precipitation (MAP) was the strongest correlation of the Ca:Mg ratio.

For *P. hwangshanensis*, Ca concentration had no significant correlation with all factors. As for Mg concentration, it was clearly correlated with MAT as *P. massoniana*. Ca:Mg ratio had a significant and strong correlation with longitude, latitude, and MAT. It was found that for both species, Mg concentration was only significantly correlated to MAT. Ca:Mg ratio of the two species were significantly correlated to longitude and MAT.

## 3. Discussion

### 3.1. Concentration of Calcium and Magnesium

The leaf is the most active organ of plant metabolism, and its chemical element concentration can reflect the characteristics of the absorption and accumulation of elements by plants [52]. More importantly, it can indicate the adaptive strategies of plants to different environments. In turn, the environment can also affect the concentration of nutrient elements in plants. An important cause of the difference in foliage Ca and Mg concentration is tree species. The value for *P. canariensis* and *P. halepensis* seemed to be much higher than that for *P. taeda* in both Ca and Mg (Table 3). Within a species, Ca and Mg nutrition may vary depending on region or site (Table 4). Ca and Mg concentration generally decreased with tree age [53]. Similarly, Ca concentration in *P. pinaster* and *P. radiata* decreased significantly with needle age; this difference, however, did not appear in Mg [54]. It was worth pointing out that the concentrations of two elements in young needles were usually lower than those in mature needles [39]. Ca concentration was lower in green needles than that in the litter, while Mg concentration was the opposite [55]. Elevated Ca and Mg concentrations for *P. halepensis* were found in winter [56]. In addition to tree age and needle development stage, environmental factors also affected the nutrient levels of these two elements. Significantly higher foliar Ca concentration in Aleppo pine could be explained by the nature of the calcareous soil parent material [42]. Ca and Mg concentrations in densely populated areas decreased [57]. Silvicultural treatments reduced foliage Ca and Mg concentrations [58]. However, another study showed that thinning treatments did not change foliage Ca and Mg concentrations in *P. halepensis* [59].

### 3.2. Correlation between Calcium and Magnesium

Different populations had similar patterns of correlation between these two elements. The correlation between Ca and Mg concentration was positive. Especially the correlations of *P. massoniana* in Mt. Lushan and Mt. Jinggangshan and of *P. hwangshanensis* in Mt. Jinggangshan were significant. It suggested that these two elements promote each other’s absorption. The same result was confirmed, and it showed that there was no antagonistic effect of Ca concentrations in soils on Mg uptake [42]. Calcium and magnesium have important effects on various vital movements of cells. Cellular functions of Ca^2+^ are structural and act as a secondary messenger, which plays a role in biotic and abiotic stress, stomatal regulation, and physical damage [12,67]. The majority of cellular Mg^2+^ has roles as enzyme cofactors and in the stabilization of nucleotides and nucleic acids [1].

In the current study, we could not verify the existence of the ‘structure and photosynthetic activity axis’ for foliage Ca and Mg nutrients in pines, but as stated above, it was found that Ca and Mg concentrations were related. This correlation is also reflected in the Ca:Mg ratio. Ca:Mg ratio was recognized as one of the key indicators for plant nutrient status [68]. According to previous reports, the foliage Ca:Mg ratio in plants is plastic within a certain range (general plants 1.0~6.67 [58]; trees 2.78~14.47, shrubs 3.47~6.48, grasses 2.69~3.84 [68]). Compared with the references, the foliage Ca:Mg ratio of *P. massoniana* (1.10~9.11) and *P. hwangshanensis* (1.89~8.73) is roughly within this range, but the minimum value is outside this range due to a series of complex factors. Although the issue of the ‘structure and photosynthetic activity axis’ still needs empirical studies, we can find some clues related to this topic. The highest concentrations of Ca and Mg were measured in leaf palisade and spongy mesophyll cells, respectively, and this conservative mechanism of foliage Ca and Mg distribution may be related to Ca and Mg homeostasis in plants [69]. For the regulation of Ca and Mg homeostasis in plants, a family of CorA-type Mg^2+^ transporters and related signal networks have been reported recently [70].

### 3.3. Genetic and Environmental Variation of Calcium and Magnesium

Different traits may have different sources of variation, mainly including genetic and environmental factors. Mg concentration was constrained more strongly genetically than environmentally, with 0.27 of the variance apportioned to species. Only 0.11 was due to elevation, which represented environmental effects partly. The variation of Ca concentration was greatly affected by elevation, with 0.58 of the total variance. The trait varied due to different environments and had a much higher level of plasticity. Intra-species variability was an apparent influencing factor in the variation of Ca and Mg concentration, as well as the Ca:Mg ratio. All traits had a certain proportion among individuals, consistent with previous studies [71]. There are also other reasons for variation. The functional type showed the greatest direct influence on most leaf minerals in the research of a larger research scale [72]. Therefore, the results of the source of variation for different circumstances are not in complete accord.

All element concentrations of the two species except the Ca concentration of *P. hwangshanensis* had significant differences among populations. Actually, the population could be regarded as the result of a complex interaction between genetic variation and environmental factors. Species in different sites may have genetic variation due to geographical isolation. In addition, different specific environments, together with the former, probably lead to differences in plant traits. However, there was no significant difference in the Ca concentration of *P. hwangshanensis*, which may be the result of the lack of randomness and extensiveness of sampling.

Element concentration is affected by various environmental factors. Foliar nutrient concentration might be the result of a complex interaction between soil nutrients and effective availability caused by climate, water, and other site and treatment effects [58]. Previous studies identified Ca concentration of pine needles was significant relative to elevation [73]. In our study, the Ca concentration of *P. massoniana* also displayed a positive and significant correlation with elevation. Elevation can cause changes in temperature, light, moisture, and other habitat factors, thus affecting plant growth and physiological metabolism [74,75].

Ca concentration of *P. massoniana* was significantly correlated with longitude and latitude. Leaf minerals exhibited significant latitudinal and longitudinal trends, which meant they had similar environmental and biological controls that shaped the biogeographic patterns of the plant minerals [72]. The concentrations of plant elements reflect the adaptation to changeable conditions from the north to south and from west to east in China.

MAT had a significant effect on Ca and Mg concentration as well as the Ca:Mg ratio of *P. massoniana*. And the strongest relationship was with Ca concentration. Meanwhile, the Mg concentration of *P. hwangshanensis* was only significantly related to MAT. Temperature and precipitation can further affect the element concentration of leaves by affecting both plant growth rate and soil nutrients [76]. However, most traits in our study had no significant relationship with MAP. It was speculated that the relationship between element concentration and the environment was weakened after data was processed.

Nutrient concentration in tree species has been used to assess the nutrient status of forests [56]. Leaf nutrient concentrations can also be used as an indicator of stand development and health status [58]. The cutting of forests has taken away branches, leaves, and other parts with higher reserves of nutrient elements, resulting in a large number of nutrient element losses. The needle nutrient concentrations for most of the nutrients studied except for Na, Mn, and C could not be affected after eight years of thinning [59]. So, we should choose scientific and reasonable methods for logging to minimize the impact of human activities on forest nutrients. Otherwise, understanding the variation patterns of nutrient elements can be crucial to planning the best silvicultural practices as a basic strategy for improving stand resilience.

## 4. Materials and Methods

### 4.1. Plant Sampling

The samples were taken from five different sites in China, including Mt. Sanqingshan, Mt. Lushan, Mt. Jinggangshan, Mt. Huangshan, and Mt. Tianmushan. All these study sites need to meet the following conditions: (1) with both *P. massoniana* and *P. hwangshanensis* populations; (2) with an elevation gradient; (3) with both *P. massoniana* and *P. hwangshanensis* as a dominant species in local communities, respectively. These places are located in the middle subtropical zone, and the landforms are characterized by middle and low mountains, hills, valleys, and plains. It has an obvious monsoon climate, characterized by hot summer and warm winter, four distinct seasons, rain and heat over the same period, and developed monsoon. The annual precipitation is generally abundant, mostly 1000~2000 mm. The average annual temperature is usually about 16~20 °C, while the average temperature of the coldest month is generally between 5~10 °C. The typical soil is mainly red or yellow soil. The zonal vegetation is evergreen, broad-leaved forest with warm and temperate coniferous forests.

**Figure 3:**
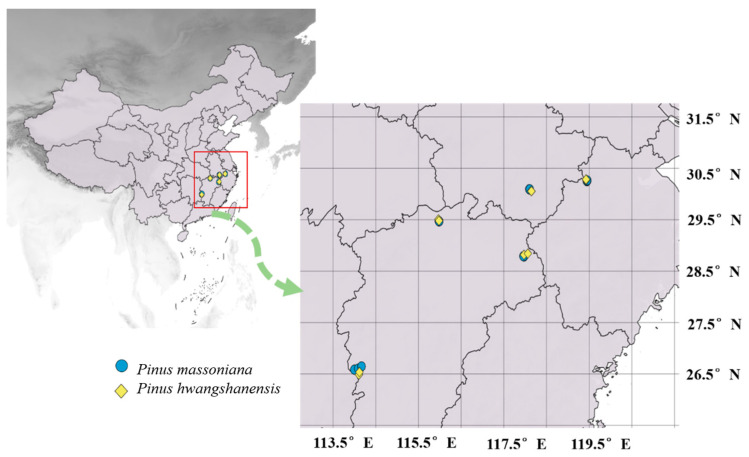
Distribution of the sampling points. Blue icons stand for *P. massoniana*, yellow icons stand for *P. hwangshanensis*.

*P. hwangshanensis* is one of the representative coniferous tree species in the eastern subtropical mountains of China. It is barren tolerant and distributed in high-elevation areas. *P. massoniana* is an important afforestation tree species in barren mountains in China, usually distributed below 700 m above sea level. To obtain samples of the two pines, the sample plot with an area of 20 × 50 m was established before sampling. For *P. massoniana*, the plots were selected at different elevations below 600 m. Besides, roughly consistent schemes were used for *P. hwangshanensis*, but the selected elevation was above 800 m (Table 4, Figure 3). Three healthy and well-growing individuals were chosen randomly and as scattered as possible in each plot. Foliar samples on annual branches were collected from the outer layer of the lower crown using a pruning pole and stored in plastic bags with silica gel to keep dry.

### 4.2. Chemical Analyses

The needles were washed in deionized water, dried at the temperature of 80 °C, and sieved through a sieve with a mesh diameter of 0.3 mm after being homogenized in a laboratory grinder. The powder samples were digested in a mixture of HNO_3_ and HCIO_4_ = 4:1. Next, the concentrations of Ca and Mg were measured by inductively coupled plasma optical emission spectrometry (ICP-OES).

### 4.3. Environmental Variables

Leaf nutrient elements are closely related to environmental factors, including temperature, precipitation, elevation, and others. Mean annual temperature (MAT) and mean annual precipitation (MAP) were obtained from the website (http://www.worldclim.org/), accessed on 20 September 2022. In addition, elevation, longitude, and latitude were recorded when sampling.

### 4.4. Statistical Analyses

In order to compare the concentration of calcium and magnesium, the calcium-magnesium ratio of two species from five populations, mean, minimum and maximum values, and other results were calculated. One-way ANOVA and multiple comparisons were made with populations as the main factors. The difference between species was also obtained through one-way ANOVA. All the data were transformed before analysis for better data normality. Pearson correlation analysis was taken to understand the correlation between Ca and Mg concentration. All analyses were conducted with the statistical software SPSS 27.

A multilevel model was fitted for each trait about species, populations, elevation, and ‘within’. The ‘within’ was regarded as natural intra-species variability within the plot and all variables were log-transformed before analysis. All parameters estimated by the REML module of the R statistical package were nested in the specified order to partition the variance to different levels [71]. It may be conducive to compare and analyze the sources of variation of these variables.

A Mantel test measures the correlation between two matrices typically containing measures of distance. In our study, trait variables and environmental variables were transformed into matrices. Computing the correlation and significance between trait variables and environmental variables through the Mantel test with the package ‘linkET’ in R 4.2.1. Meanwhile, Spearman’s correlation coefficients were calculated between environmental variables. The Mantel composite graph was visualized by ‘ggplot2’ in R [77].

## 5. Conclusions

Our study systematically investigated the concentrations of calcium and magnesium for *P. massoniana* and *P. hwangshanensis* in different sites. The concentrations of Ca and Mg had significant variations among different populations. The variation of Ca was constrained more strongly environmentally than genetically, while Mg was the opposite. Concentrations of both elements had a certain correlation with longitude, latitude, MAT, and elevation. Our study on the variation pattern of calcium and magnesium elements can provide a basis for afforestation management and forest nutrient protection. Besides, the soil nutrient conditions at the sampling sites were not measured. The relationship between soil nutrition and leaf nutrition is not clear, so further research is needed.

## Figures and Tables

**Table 1:** Ca and Mg concentrations in the needles of *P. massoniana* and *P. hwangshanensis* among populations (mg·g^−1^).

Ca	*P. massoniana*					*P. hwangshanensis*				
Population	H	J	L	S	T	H	J	L	S	T
Mean	4.00 ab	4.01 b	2.59 b	5.62 a*	3.39 b	3.78 a	2.85 a	3.11 a	2.97 a	3.42 a
Median	3.53	4.27	2.62	5.48	3.13	3.31	2.95	3.28	3.08	3.40
Variance	3.96	5.06	0.28	0.65	1.38	1.77	0.42	0.38	0.91	0.76
SD	1.99	2.25	0.53	0.81	1.18	1.33	0.65	0.61	0.95	0.87
Minimum	1.74	1.10	1.49	4.73	1.77	2.18	1.57	1.91	1.88	2.46
Maximum	8.38	8.22	3.53	7.08	5.09	5.94	3.62	3.81	4.26	5.19
CV	49.77%	56.08%	20.60%	14.38%	34.72%	35.18%	22.71%	19.74%	32.05%	25.41%
Mg	*P. massoniana*					*P. hwangshanensis*				
Population	H	J	L	S	T	H	J	L	S	T
Mean	0.92 c*	1.41 a*	1.07 bc	1.13 abc	1.32 ab*	0.67 c	1.09 a	1.00 ab	0.87 abc	0.84 bc
Median	0.92	1.37	0.93	1.02	1.38	0.68	1.13	0.92	0.79	0.89
Variance	0.05	0.14	0.07	0.07	0.05	0.02	0.02	0.09	0.05	0.05
SD	0.23	0.38	0.27	0.27	0.21	0.13	0.15	0.31	0.23	0.22
Minimum	0.60	0.92	0.73	0.90	0.98	0.45	0.83	0.72	0.69	0.44
Maximum	1.28	2.23	1.48	1.48	1.53	0.82	1.32	1.77	1.27	1.12
CV	24.62%	26.85%	25.33%	23.74%	16.20%	19.19%	13.38%	30.89%	26.13%	26.09%
Ca:Mg	*P. massoniana*					*P. hwangshanensis*				
Population	H	J	L	S	T	H	J	L	S	T
Mean	4.33 a	2.75 b	2.47 b*	5.20 a*	2.56 b*	5.65 a	2.60 c	3.23 c	3.45 bc	4.22 b
Median	3.52	2.99	2.39	4.89	2.27	5.27	2.74	3.16	3.34	4.22
Variance	3.85	1.59	0.20	2.11	0.67	2.84	0.17	0.53	0.88	1.17
SD	1.96	1.26	0.44	1.45	0.82	1.69	0.41	0.73	0.94	1.08
Minimum	2.92	1.10	1.86	3.87	1.68	3.79	1.89	2.15	2.30	3.00
Maximum	9.11	4.83	3.08	7.77	4.28	8.73	3.05	4.33	4.48	5.94
CV	45.34%	45.79%	17.99%	27.96%	32.05%	29.85%	15.71%	22.55%	27.22%	25.62%

SD = Standard Deviation, CV = Coefficient of Variance; S = Mt. Sanqingshan, L = Mt. Lushan, J = Mt. Jinggangshan, H = Mt. Huangshan, T = Mt. Tianmushan. Different lowercase letters (abc) after the numerical value indicate that the difference between populations reaches a 0.05 significant level. * It indicates that there is a significant difference between the two pines in the population.

**Table 2:** Pearson correlation coefficients between calcium and magnesium concentrations in the needles of *P. massoniana* and *P. hwangshanensis*.

Population	S	L	J	H	T
*P. hwangshanensis*	0.664	0.512	0.792 *	0.524	0.576
*P. massoniana*	0.012	0.713 *	0.680 *	0.419	0.504

* *p* < 0.05.

**Table 3:** Foliage Ca and Mg concentrations of pine (*Pinus*) needles in previous reports.

Species	Countries and Regions	Ca Concentration (mg·g^−1^)	Mg Concentration (mg·g^−1^)
*P. elliottii*	China, Fujian	2.0	0.8 [60]
	China, Jiangxi	3.8	0.9 [61]
*P. kesiya* var. *langbianensis*	China, Yunnan	1.9	1.4 [62]
*P. massoniana*	China, Sichuan	6.8	1.3 [51]
*P. thunbergii*	China, Shandong	4.5	1.6 [63]
*P. yunnanensis*	China, Yunnan	3.2	1.4 [64]
*P. brutia*	Turkey, Istanbul	2.3~4.6	0.7~0.8 [57]
*P. canariensis*	Spain, Tenerife	9.1	1.5 [39]
*P. halepensis*	Spain, Yeste/Calasparra	7.0/7.5	2.7/2.0 [58]
	Greece, Parnitha	7.9	1.5 [42]
	Spain, Granada	2.4	0.2 [59]
	Spain, Granada	12.3	2.5 [56]
*P. pinaster*	Spain, Galicia	1.1	1.6 [54]
*P. radiata*	Spain, Galicia	3.6	0.9 [54]
*P. sylvestris*	Finland, Suonenjoki	4.3	0.5 [55]
	Lithuania, Neris	4.9	1.2 [43]
*P. taeda*	USA, 11 Southern States	1.8	1.0 [65]
	USA, Texas	2.8~4.0	1.8~2.3 [53]
	USA, 8 southeastern States	1.7	1.0 [41]
	Brazil, Southern region	1.8	0.5 [66]

Numbers in the square frame in the last column correspond to the references cited.

**Table 4:** The geographic locations of sampling populations in *P*. *massoniana* and *P. hwangshanensis*.

Population	Abbreviation	Longitude (°E)	Latitude (°N)	Site (plot) and Elevation (m)	
				*P. massoniana*	*P. hwangshanensis*
Mt. Sanqingshan, Jiangxi	S	118°00′~118°06′	28°52′~28°57′	300, 400	1062, 1500
Mt. Lushan, Jiangxi	L	115°50′~116°10′	29°28′~29°45′	300, 400, 500	900, 1000, 1100
Mt. Jinggangshan, Jiangxi	J	113°39′~114°23′	26°27′~26°40′	300, 500, 600	900, 1100, 1300
Mt. Huangshan, Anhui	H	116°49′~118°59′	29°13′~31°05′	350, 450, 550	900, 1100, 1200
Mt. Tianmushan, Zhejiang	T	118°36′~120°06′	29°52′~30°55′	340, 480, 580	1200, 1400, 1500

## Data Availability

All data supporting the findings of this study are available within the paper, which are published online.
